# LncRNA BLACAT1 May Serve as a Prognostic Predictor in Cancer: Evidence from a Meta-Analysis

**DOI:** 10.1155/2019/1275491

**Published:** 2019-04-02

**Authors:** Hongyan Lu, Haoran Liu, Xiaoqi Yang, Tao Ye, Peng Lv, Xiaoliang Wu, Zhangqun Ye

**Affiliations:** ^1^Department of Urology, Tong Hospital, Tongji Medical College, Huazhong University of Science and Technology, Wuhan 430030, China; ^2^Hubei Institute of Urology, Wuhan 430030, China

## Abstract

**Background:**

As a newly discovered lncRNA, bladder cancer-associated transcript 1 (BLACAT1) has been reported to correlate with poor clinical outcomes in several different cancers. This study aimed to evaluate its generalized predictive value for cancer prognosis.

**Materials and Methods:**

We thoroughly searched PubMed, Embase, and Web of Science databases for eligible studies published until November 11, 2018, in which the relationship between BLACAT1 expression and cancer prognosis was explored. The analyses were performed using Review Manager Version 5.3 and Stata SE 12.0. The primary endpoints included overall survival (OS), pathological characteristics (TNM stage and tumor grade), lymph node metastasis (LNM), and distant metastasis.

**Results:**

Ten studies containing 861 patients with 7 different cancerous diseases were eventually included. The results demonstrated that patients with high lncRNA BLACAT1 expression had a significantly shorter OS (HR: 1.82, 95% CI: 1.44-2.30, p < 0.00001) than patients with low lncRNA BLACAT1 expression. Moreover, elevated BLACAT1 expression was significantly associated with advanced TNM stage (OR: 2.29, 95% CI: 1.15-4.56, p = 0.005), high tumor grade (OR: 1.67, 95% CI: 1.11-2.53, p = 0.01), and lymph node metastasis (OR: 2.53, 95% CI: 1.80-3.57,* p* < 0.00001). Meanwhile, the expression of BLACAT1 had no significant association with age (*p* = 0.92), gender (*p* = 0.55), and smoking (*p* = 0.62).

**Conclusion:**

High expression of lncRNA BLACAT1 may predict a poor prognosis in OS, TNM stage, tumor grade, and LNM. Its predictive roles were not significantly affected by age, gender, or smoking. Therefore, lncRNA BLACAT1 may serve as a promising predictor in cancer prognosis.

## 1. Introduction

In recent years, malignant tumors have become a serious public health problem and one of the leading causes of morbidity and mortality worldwide. The overall cancer incidence and mortality rate in the United States and China have been increasing in the past few years [1, 2]. Plenty of studies have explored the mechanism and development of cancers. Although numerous progress has been achieved in their diagnosis and treatment, the 5-year survival rate remains relatively low in most patients with cancer [1].

Long noncoding RNAs (lncRNAs) are transcripts comprising more than 200 nucleotides but not coding proteins [3]. Studies have suggested that lncRNAs play important roles in a wide variety of biological processes among cancerous cells, e.g., proliferation, invasion, apoptosis, and metabolism [4, 5]. Furthermore, numerous studies have indicated that many lncRNAs might be involved in oncogenesis and tumor-suppression [6]. Several lncRNAs (such as MALAT1, HOTAIR, and GAS5) were reported to be critical players in the transcriptional regulation of various cancer-related genes [7–9]. Finally, many lncRNAs have been reported to serve as potential therapeutic targets, independent prognostic predictors, or valuable biomarkers in cancers [10–12].

LncRNA bladder cancer-associated transcript 1 (BLACAT1, also named as linc-UBC1), on the locus of human chromosome 1q32.1, was initially identified in bladder cancer [13]. However, studies have indicated that lncRNA BLACAT1 is also overexpressed in other cancers, e.g., gastric cancer [14], small-cell lung cancer [15], and colorectal cancer [16]. LncRNA BLACAT1 could promote the proliferation, migration, and invasion of cancerous cells. Consequently, cancer patients with high lncRNA BLACAT1 expression tend to have a poor prognosis.

However, the generalized applicability of lncRNA BLACAT1 in predicting prognosis for cancers remains unknown. Herein, a meta-analysis was performed to comprehensively explore the relationship between prognosis and lncRNA BLACAT1 expression in different cancers.

## 2. Materials and Methods

### 2.1. Search Strategy and Literature Selection

This study was performed according to the Preferred Reporting Items for Systematic Reviews and Meta-Analysis statement. Eligible literature was searched through PubMed, Embase, and Web of Science databases to November 11, 2018. The following keywords were used in combination for searches: (“long non-coding RNA” OR “lncRNA”), (“BLACAT1” OR “linc-UBC1” OR “bladder cancer-associated transcript 1”), (“carcinoma” OR “cancer” OR “neoplasm”), AND (“clinical outcome” OR “prognosis” OR “survival”). The reference lists of included articles were also screened for potentially missing literature.

### 2.2. Inclusion and Exclusion Criteria

All the eligible studies were assessed for inclusion or exclusion by 2 authors independently. Controversies between them were resolved via negotiation. Studies were included if they evaluated the prognostic value of lncRNA BLACAT1 in patients with any type of cancer. The survival outcomes were reported as OS with hazard ratios (HR) and corresponding 95% confidence intervals (CI), or these data could be extrapolated through the survival curve. Studies were excluded if they were (1) non-English papers, (2) non-human studies, (3) reviews, letters, or case reports, or (4) lack of available survival data.

### 2.3. Data Extraction and Quality Assessment

Two authors screened each eligible study and extracted the essential information independently, including the name of first author, year of publication, origin country, cancer type, tumor stage, sample size, type of specimen, method for detecting BLACAT1, cut-off value, HR, and corresponding 95% CI for OS, as well as clinical features and pathological characteristics. HR from multivariable analysis had priority to be chosen when available. For studies only representing the Kaplan-Meier curve, a method described by* Tierney* et al. was used to obtain prognostic data indirectly [19]. The Newcastle-Ottawa Scale (NOS) was used to evaluate the quality of the included studies [20].

### 2.4. Statistical Analysis

HR and corresponding 95% CI for OS was used to determine the pooled effect. Regarding pathological characteristics, TNM stages I and II were combined and III and IV were combined; tumor grades G2 and G3 were also combined. The odds ratio (OR) was applied as the outcome estimation for data pooling.

Heterogeneity among the included studies was analyzed by the I^2^ test and *χ*2-based Q test. I^2^ < 50% and P > 0.05 meant no significant heterogeneity across the studies; therefore a fixed-effect model was used. I^2^ > 50% and p < 0.05 denoted strong heterogeneity; finally a random-effect model was applied. Funnel plots were utilized to assess potential publication bias. All p values were two-tailed, and p values < 0.05 were defined as statistical significance. Review Manager 5.3 (Cochrane Collaboration, London, UK) and Stata SE 12.0 (StataCorp LLC, College Station, Texas, USA) were used to perform all the analyses. The impacts of lncRNA BLACAT1 expression on OS, clinical features, and pathological characteristics were considered statistically significant if the 95% CI for the combined HR (or OR) did not cross 1.

## 3. Results

### 3.1. Characteristics of Eligible Studies

**Figure 1:**
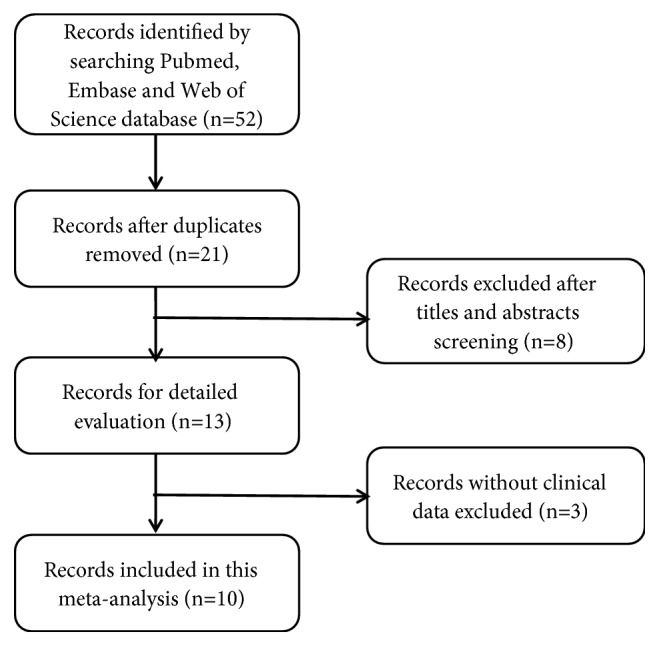
Flow diagram of the literature selection.

The process of literature selection was detailed in Figure 1. A total of 52 records were retrieved to November 11, 2018. After excluding the duplicated and unqualified papers, 10 studies involving 861 patients with 7 different types of cancers were included. These studies comprised lung cancer [12, 16], urothelial carcinoma [10, 19], colorectal cancer [13, 20], gastric cancer [11], papillary thyroid cancer [21], esophageal squamous cell carcinoma [22], and cervical cancer [23].

The characteristics of the included studies were summarized in Table 1. Of the 10 studies, 1 and 9 were conducted in Germany and China, respectively, published from 2013 to 2018. The sample size of the included studies ranged from 48 to 133, and 4 studies enrolled over 100 participants each. LncRNA BLACAT1 expression was measured by quantitative real-time PCR in all studies. Based on the expression level, patients in these studies were divided into 2 groups, namely, high and low lncRNA BLACAT1 expression groups. Of the 9 studies reported the HR and corresponding 95% CI for OS, 5 had explicit available data on OS; however, in 4 of them, data on OS were extrapolated through survival curve indirectly. Furthermore, 7 studies represented the relationship between lncRNA BLACAT1 expression and clinicopathological features, including age, gender, smoking, TNM stage, tumor grade, lymph node metastasis (LNM), and distant metastasis (DM). The quality of the included studies was confirmed using Newcastle-Ottawa Scale (NOS).

### 3.2. Association between LncRNA BLACAT1 and OS

**Figure 2:**
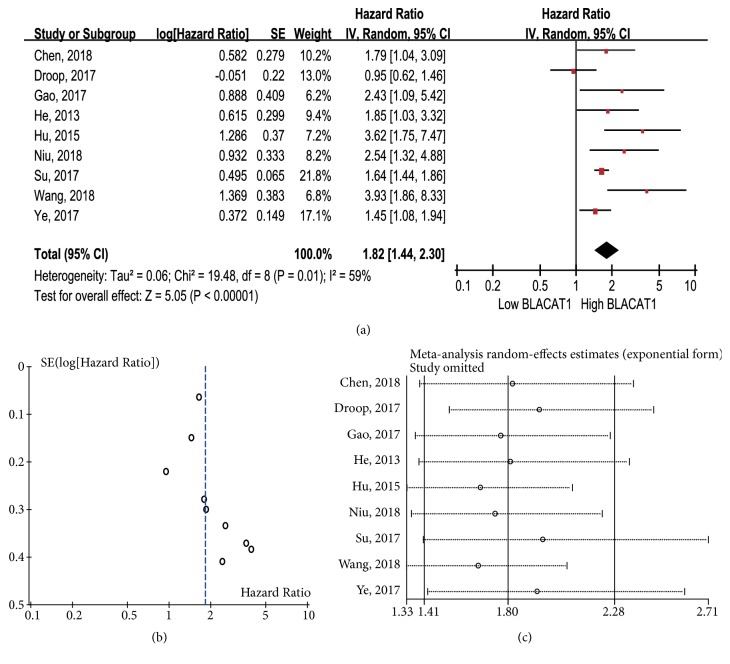
(a) Forest plot for the meta-analysis of OS; (b) funnel plot for the meta-analysis of OS; (c) sensitivity analysis for the meta-analysis of OS.

The HR from 9 eligible studies (including 774 patients) was combined to determine the association between lncRNA BLACAT1 expression and OS. As shown in Figure 2(a), strong heterogeneity was observed among the studies (I^2^ = 59%,* p* = 0.01); therefore a random-effect model was used. The combined HR was 1.82 (95% CI: 1.44-2.30,* p* < 0.00001), suggesting that the high lncRNA BLACAT1 expression group had significantly poorer OS than the low lncRNA BLACAT1 expression group. No obvious publication bias was detected in the studies according to visual assessment of funnel plot (Figure 2(b)) and Egger' test (*p* = 0.102, data not shown). Furthermore, sensitivity analysis demonstrated little influence of individual study on our final results (Figure 2(c)).

**Figure 3:**
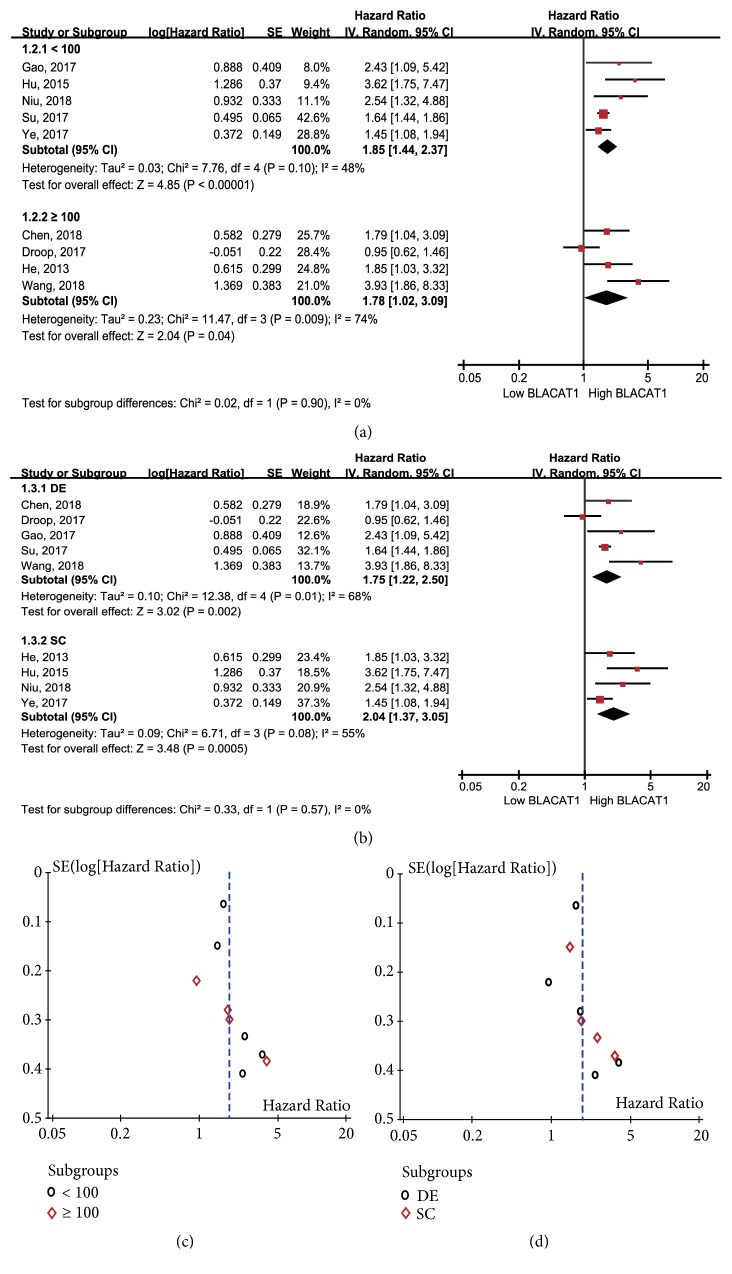
Forest plots of the subgroup analysis of OS based on (a) sample size and (b) the obtained methods of HR. Funnel plots of the subgroup analysis of OS based on (c) sample size and (d) the obtained methods of HR.

The subgroup analysis of OS was also performed according to the sample size, the method to obtain HR and 95% CI for OS. Results showed that high lncRNA BLACAT1 expression could predict low OS regardless of the scale of sample size, < 100 patients (*p* < 0.00001) or ≥ 100 patients (*p* = 0.04, Figure 3(a)). With respect to the methods to obtain HR and 95% CI for OS, similar results were achieved in the data extrapolated subgroup (HR: 1.75, 95% CI: 1.22-2.50,* p* = 0.002, Figure 3(b)) and the survival curve subgroup (HR: 2.04, 95% CI: 1.37-3.05,* p* = 0.0005, Figure 3(b)). No obvious bias was observed in the analysis on the basis of the funnel plots (Figures 3(c) and 3(d)).

### 3.3. Association between LncRNA BLACAT1 and Pathological Characteristics

There was significant difference between elevated lncRNA BLACAT1 expression and advanced tumor TNM stage (OR: 2.29, 95% CI: 1.15-4.56,* p* = 0.005, I^2^ = 70%, random-effect, Figure 5(a)) and high tumor grade (OR: 1.67, 95% CI: 1.11-2.53,* p* = 0.01, I^2^ = 70%, fixed-effect, Figure 5(b)). Unfortunately, we failed to investigate the relationship between lncRNA BLACAT1 expression and other pathological characteristics due to insufficient data.

### 3.4. Association between LncRNA BLACAT1 and Metastasis

Seven and two eligible studies reported the states of LNM and DM, respectively, based on the lncRNA BLACAT1 expression levels. Compared with patients with low BLACAT1 expression, patients with high lncRNA BLACAT1 expression had significantly higher risk of LNM (OR: 2.53, 95% CI: 1.80-3.57,* p* < 0.00001, I^2^ = 15%, fixed-effect, Figure 6(a)). Only 2 studies reported the correlation between BLACAT1 expression and DM, the pooled HR was 2.39, but the 95% CI overlapped 1 (95% CI: 0.85-6.75), and high heterogeneity was observed between the studies (I^2^ = 52%, Figure 6(b)). Therefore, it was invalid to determine the relationship between lncRNA BLACAT1 expression and DM.

### 3.5. Association between LncRNA BLACAT1 and Clinical Features

**Figure 4:**
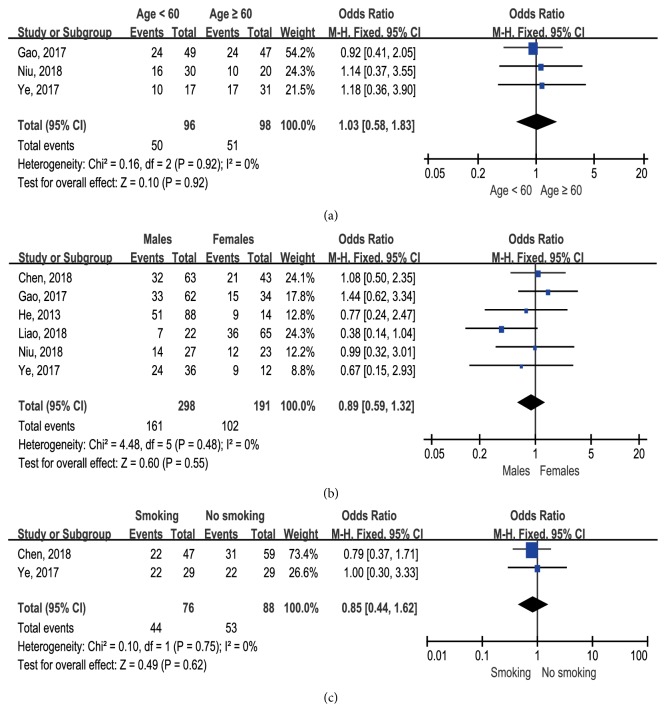
Forest plots showing the association between lncRNA BLACAT1 expression and clinical features: (a) age; (b) gender; (c) smoking.

**Figure 5:**
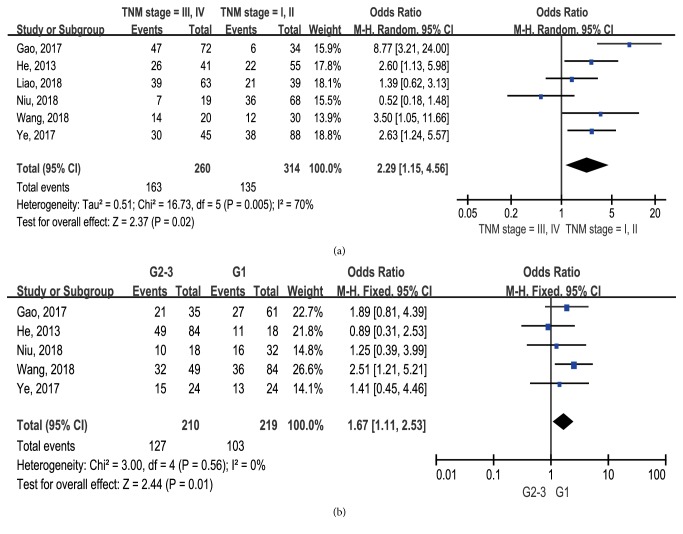
Forest plots showing the association between lncRNA BLACAT1 expression and pathological features: (a) TNM stage; (b) tumor grade.

**Figure 6:**
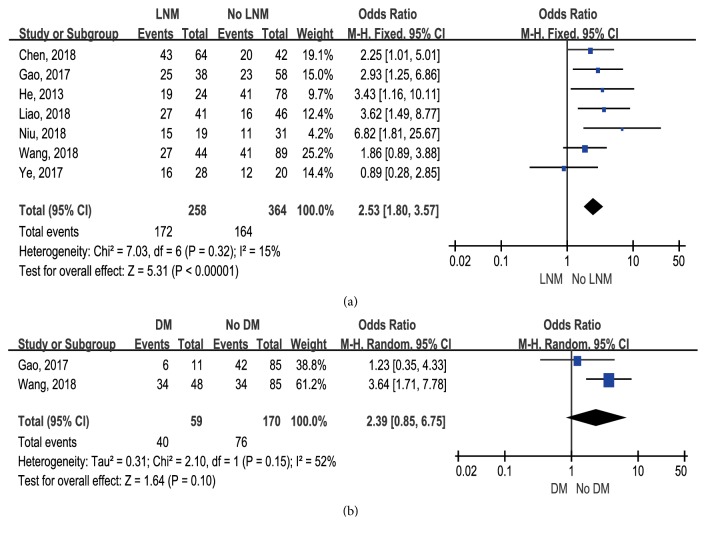
(a) Forest plot for the association between lncRNA BLACAT1 and lymph node metastasis. (b) Forest plot for the association between lncRNA BLACAT1 and distant metastasis.

The relationship between lncRNA BLACAT1 expression and age, gender, and smoking was investigated by 3, 6, and 2 studies, respectively. Results indicated that lncRNA BLACAT1 expression was not significantly correlated with age (*p* = 0.92, Figure 4(a)), gender (*p* = 0.55, Figure 4(b)), and smoking (*p* = 0.62, Figure 4(c)).

## 4. Discussion

Accumulating evidence has proven that lncRNA BLACAT1 is closely related to cancer. Initially, lncRNA BLACAT1 was identified as a frequently overexpressed oncogene in bladder cancer tissues. Its overexpression is associated with LNM and poor prognosis in patients with bladder cancer [13]. Currently, lncRNA BLACAT1 have been confirmed as a dysregulated oncogene in other several malignancies, e.g., lung cancer, colorectal cancer, gastric cancer, papillary thyroid cancer, esophageal squamous cell carcinoma, and cervical cancer. Moreover, lncRNA BLACAT1 knockdown significantly inhibited cell motility, proliferation, and invasion [21, 23, 24]. Due to its oncogenic potential, lncRNA BLACAT1 is defined as a carcinogenic lncRNA in many kinds of cancers. Recently, many researchers focused on lncRNA BLACAT1 due to its potential role in predicting cancer prognosis. However, controversies emerged regarding the predictive value of lncRNA BLACAT1 in some prognostic parameters, e.g., TNM stage, tumor grade, and LNM.

Our results indicated that patients with high lncRNA BLACAT1 expression tended to have poorer OS than those with low BLACAT1 expression. Namely, high lncRNA BLACAT1 expression may serve as a negative predictor for cancer prognosis. Meanwhile, this study also demonstrated that high BLACAT1 expression significantly correlated with more advanced TNM stage, higher tumor grade, and higher risk of LNM. All these findings indicated that lncRNA BLACAT1 could be a potential predictor of worse clinical outcomes for cancer patients. However, the underlying molecular mechanisms of high lncRNA BLACAT1 expression associated with worse prognosis remain elusive.

Many studies have investigated the functional mechanisms of lncRNA BLACAT1 on tumorigenesis and progression in various cancers. Wnt/*β*-catenin signal pathway has been confirmed to be involved in the regulation of cell proliferation, migration, and invasion in certain cancers [25, 26].* Wang* et al. reported that knockdown of lncRNA BLACAT1 might inhibit the proliferation, migration, and invasion of cervical cancer cells by suppressing the activation of Wnt/*β*-catenin signal pathway [24]. Cdk inhibitors (CKIs) may regulate the kinase activity of Cdk/cyclin complexes and generally function as tumor suppressors.* Su* et al. identified that lncRNA BLACAT1 was a critical player in the cell cycle of colorectal cancer via modulating the expression of P15 (a member of CKIs family) [23]. Furthermore, a study by* Ye* et al. demonstrated that lncRNA BLACAT1 may promote the proliferation, migration, and invasion of non-small-cell lung cancer cell by sponging miR-144 [21], which is an important member of tumor-suppressive microRNAs [27, 28]. Otherwise, several studies also have reported that lncRNA BLACAT1 may be involved in chemoresistance.* Huang* et al. indicated that lncRNA BLACAT1 could regulate autophagy and promote chemoresistance of non-small-cell lung cancer cells via miR-17/ATG7 axis [29].* Wu* et al. demonstrated that BLACAT1 promoted the oxaliplatin-resistance of gastric cancer through BLACAT1/miR-361/ABCB1 regulatory pathway, providing a brand new insight for the oxaliplatin-resistance of gastric cancer [30]. All the findings concluded that lncRNA BLACAT1 might be a promising therapeutic target for cancers.

Our results further revealed that the level of lncRNA BLACAT1 expression was a valuable and potential prognostic predictor for cancers. However, our study has several limitations. First, the cut-off value of lncRNA BLACAT1 overexpression was not uniform among the included studies. Second, the number of studies and the corresponding sample size involved in each study were relatively small. Third, no sufficient data could be used to explore the prognostic value of lncRNA BLACAT1 expression in a specific type of cancer. Finally, most patients included in this research were from China, which might weaken the generalization of our results for other ethnic populations.

In conclusion, our study suggests that high lncRNA BLACAT1 expression is a valuable predictor for poor cancer prognosis in OS, TNM stage, tumor grade, and LNM. Expression of lncRNA BLACAT1 is not associated with age, gender, and smoking. Meanwhile, its predictive values are not like to be affected by age, gender, and smoking. Therefore, lncRNA BLACAT1 is a promising prognostic biomarker for various cancers. However, more comprehensive, larger-scale, and highly qualified multicenter studies are still required to confirm its predictive value in various cancers further.

## Figures and Tables

**Table 1:** Characteristics of studies included in the meta-analysis.

Study (year)	Country	Cancer type	Stage	Sample size	BLACAT1		Specimens	Method	Cut-off	Outcome	HR availability	NOS
					High	Low						
Chen, 2018	China	Small‐cell lung cancer	I-IV	106	53	53	Tissue	qRT‐PCR	Median	OS	DE	8
Droop, 2017	Germany	Urothelial carcinoma	I-IV	106	53	53	Tissue	qRT‐PCR	Median	OS	DE	7
Gao, 2017	China	Colorectal cancer	I-IV	96	48	48	Tissue	qRT‐PCR	Median	OS	DE	8
He, 2013	China	Urothelial carcinoma	I-IV	102	60	42	Tissue	qRT‐PCR	FC >1.5	OS	SC	7
Hu, 2015	China	Gastric cancer	I-IV	85	32	53	Tissue	qRT‐PCR	Median	OS	SC	6
Liao, 2018 [17]	China	Papillary thyroid cancer	I-IV	87	43	44	Plasma	qRT‐PCR	Median	NR	NR	7
Niu, 2018 [18]	China	Esophageal squamous cell carcinoma	I-IV	50	26	24	Tissue	qRT‐PCR	NR	OS	SC	7
Su, 2017	China	Colorectal cancer	I-IV	48	24	24	Tissue	qRT‐PCR	Median	OS	DE	8
Wang, 2018	China	Cervical cancer	I-IV	133	68	65	Tissue	qRT‐PCR	Median	OS	DE	8
Ye, 2017	China	Non-small-cell lung cancer	I-III	48	28	20	Tissue	qRT‐PCR	NR	OS	SC	7

HR, hazard ratio; NOS, Newcastle-Ottawa Scale; qRT‐PCR, quantitative real time-polymerase chain reaction; OS, overall survival; DE, data extrapolated; FC, fold change; SC, survival curve; NR, not reported.

## References

[1] Siegel R. L., Miller K. D., Jemal A. (2017). Cancer statistics, 2017. *CA: A Cancer Journal for Clinicians*.

[2] Chen W., Zheng R., Baade P. D. (2016). Cancer statistics in China, 2015. *CA: A Cancer Journal for Clinicians*.

[3] Brockdorff N., Ashworth A., Kay G. F. (1992). The product of the mouse Xist gene is a 15 kb inactive X-specific transcript containing no conserved ORF and located in the nucleus. *Cell*.

[4] Mercer T. R., Dinger M. E., Mattick J. S. (2009). Long non-coding RNAs: insights into functions. *Nature Reviews Genetics*.

[5] Yang G., Lu X., Yuan L. (2014). LncRNA: A link between RNA and cancer. *Biochimica et Biophysica Acta (BBA) - Gene Regulatory Mechanisms*.

[6] Fatica A., Bozzoni I. (2014). Long non-coding RNAs: new players in cell differentiation and development. *Nature Reviews Genetics*.

[7] Li Z. X., Zhu Q. N., Zhang H. B. (2018). MALAT1: a potential biomarker in cancer. *Cancer Manag Res*.

[8] Bhan A., Mandal S. S. (2015). LncRNA HOTAIR: A master regulator of chromatin dynamics and cancer. *Biochimica et Biophysica Acta (BBA) - Reviews on Cancer*.

[9] Sun W., Yang Y., Xu C., Guo J. (2017). Regulatory mechanisms of long noncoding RNAs on gene expression in cancers. *Cancer Genetics*.

[10] Gupta R. A., Shah N., Wang K. C. (2010). Long non-coding RNA HOTAIR reprograms chromatin state to promote cancer metastasis. *Nature*.

[11] Chen C., Feng Y., Wang X. (2018). LncRNA ZEB1-AS1 expression in cancer prognosis: Review and meta-analysis. *Clinica Chimica Acta*.

[12] Zhang J., Yin M., Huang J. (2018). Long noncoding RNA LINC00152 as a novel predictor of lymph node metastasis and survival in human cancer: a systematic review and meta-analysis. *Clinica Chimica Acta*.

[13] He W., Cai Q., Sun F. (2013). Linc-UBC1 physically associates with polycomb repressive complex 2 (PRC2) and acts as a negative prognostic factor for lymph node metastasis and survival in bladder cancer. *Biochimica et Biophysica Acta (BBA) - Molecular Basis of Disease*.

[14] Hu Y., Pan J., Wang Y., Li L., Huang Y. (2015). Long noncoding RNA linc-UBC1 is negative prognostic factor and exhibits tumor pro-oncogenic activity in gastric cancer. *International Journal of Clinical and Experimental Pathology*.

[15] Chen W., Hang Y., Xu W. (2018). BLACAT1 predicts poor prognosis and serves as oncogenic lncRNA in small-cell lung cancer. *Journal of Cellular Biochemistry*.

[16] Gao X., Wen J., Gao P., Zhang G., Zhang G. (2017). Overexpression of the long non-coding RNA, linc-UBC1, is associated with poor prognosis and facilitates cell proliferation, migration, and invasion in colorectal cancer. *OncoTargets and Therapy*.

[17] Liao D., Lv G., Wang T. (2018). Prognostic value of long non-coding RNA BLACAT1 in patients with papillary thyroid carcinoma. *Cancer Cell International*.

[18] Niu G., Zhuang H., Li B., Cao G. (2018). Long noncoding RNA linc-UBC1 promotes tumor invasion and metastasis by regulating EZH2 and repressing E-cadherin in esophageal squamous cell carcinoma. *Journal of B.U.ON.*.

[19] Tierney J. F., Stewart L. A., Ghersi D., Burdett S., Sydes M. R. (2007). Practical methods for incorporating summary time-to-event data into meta-analysis. *Trials*.

[20] Stang A. (2010). Critical evaluation of the Newcastle-Ottawa scale for the assessment of the quality of nonrandomized studies in meta-analyses. *European Journal of Epidemiology*.

[21] Ye J., Liu L., Zheng F. (2017). Long noncoding rna bladder cancer associated transcript 1 promotes the proliferation, migration, and invasion of nonsmall cell lung cancer through sponging miR-144. *DNA and Cell Biology*.

[22] Droop J., Szarvas T., Schulz W. A. (2017). Diagnostic and prognostic value of long noncoding RNAs as biomarkers in urothelial carcinoma. *PLoS One*.

[23] Su J., Zhang E., Han L. (2017). Long noncoding RNA BLACAT1 indicates a poor prognosis of colorectal cancer and affects cell proliferation by epigenetically silencing of p15. *Cell Death Dis*.

[24] Wang C. H., Li Y. H., Tian H. L., Bao X. X., Wang Z. M. (2018). Long non-coding RNA BLACAT1 promotes cell proliferation, migration and invasion in cervical cancer through activation of Wnt/*β*-catenin signaling pathway. *European Review for Medical and Pharmacological Sciences*.

[25] Hseu Y., Lin Y., Rajendran P. (2019). Antrodia salmonea suppresses invasion and metastasis in triple-negative breast cancer cells by reversing EMT through the NF-*κ*B and Wnt/*β*-catenin signaling pathway. *Food and Chemical Toxicology*.

[26] Liu M., Sun X., Shi S. (2018). MORC2 enhances tumor growth by promoting angiogenesis and tumor-associated macrophage recruitment via Wnt/*β*-catenin in lung cancer. *Cellular Physiology and Biochemistry*.

[27] Gao Z., Zhang P., Xie M., Gao H., Yin L., Liu R. (2018). miR-144/451 cluster plays an oncogenic role in esophageal cancer by inhibiting cell invasion. *Cancer Cell International*.

[28] Yin Y., Cai J., Meng F., Sui C., Jiang Y. (2018). MiR-144 suppresses proliferation, invasion, and migration of breast cancer cells through inhibiting CEP55. *Cancer Biology & Therapy*.

[29] Huang F. X., Chen H. J., Zheng F. X. (2019). LncRNA BLACAT1 is involved in chemoresistance of non-small cell lung cancer cells by regulating autophagy. *International Journal of Oncology*.

[30] Wu X., Zheng Y., Han B., Dong X. (2018). Long noncoding RNA BLACAT1 modulates ABCB1 to promote oxaliplatin resistance of gastric cancer via sponging miR-361. *Biomedicine & Pharmacotherapy*.

